# Mine-DW-Fusion: BEV Multiscale-Enhanced Fusion Object-Detection Model for Underground Coal Mine Based on Dynamic Weight Adjustment

**DOI:** 10.3390/s25165185

**Published:** 2025-08-20

**Authors:** Wanzi Yan, Yidong Zhang, Minti Xue, Zhencai Zhu, Hao Lu, Xin Zhang, Wei Tang, Keke Xing

**Affiliations:** 1 School of Mines, China University of Mining and Technology, Xuzhou 221116, China; wzyan@cumt.edu.cn (W.Y.); tb20020032b4@cumt.edu.cn (K.X.); 2 State Key Laboratory for Fine Exploration and Intelligent Development of Coal Resources, School of Mines, China University of Mining and Technology, Xuzhou 221116, China; ydzhangcumt@126.com; 3 State Key Laboratory of Intelligent Mining Equipment Technology, School of Mechanical and Electrical Engineering, China University of Mining and Technology, Xuzhou 221116, China; xuemint@126.com (M.X.); haolucumt@163.com (H.L.); 4 School of Information and Control Engineering, China University of Mining and Technology, Xuzhou 221116, China; zhangxin_1994@126.com; 5 Artificial Intelligence Research Institute, China University of Mining and Technology, Xuzhou 221116, China; tang_wei@cumt.edu.cn

**Keywords:** autonomous driving, multimodal information fusion, environmental perception, bird’s-eye view, auxiliary transportation vehicles

## Abstract

Environmental perception is crucial for achieving autonomous driving of auxiliary haulage vehicles in underground coal mines. The complex underground environment and working conditions, such as dust pollution, uneven lighting, and sensor data abnormalities, pose challenges to multimodal fusion perception. These challenges include: (1) the lack of a reasonable and effective method for evaluating the reliability of different modality data; (2) the absence of in-depth fusion methods for different modality data that can handle sensor failures; and (3) the lack of a multimodal dataset for underground coal mines to support model training. To address these issues, this paper proposes a coal mine underground BEV multiscale-enhanced fusion perception model based on dynamic weight adjustment. First, camera and LiDAR modality data are uniformly mapped into BEV space to achieve multimodal feature alignment. Then, a Mixture of Experts-Fuzzy Logic Inference Module (MoE-FLIM) is designed to infer weights for different modality data based on BEV feature dimensions. Next, a Pyramid Multiscale Feature Enhancement and Fusion Module (PMS-FFEM) is introduced to ensure the model’s perception performance in the event of sensor data abnormalities. Lastly, a multimodal dataset for underground coal mines is constructed to provide support for model training and testing in real-world scenarios. Experimental results show that the proposed method demonstrates good accuracy and stability in object-detection tasks in coal mine underground environments, maintaining high detection performance, especially in typical complex scenes such as low light and dust fog.

## 1. Introduction

Auxiliary transportation vehicles are one of the important components in underground coal mine auxiliary haulage systems, which undertakes the transportation tasks of workers, equipment, and materials. They directly affect the production efficiency and mining security. Studying their driverless methods can solve the problems of high personnel input, low transportation efficiency, and prominent safety hazards that exist in the traditional manual driving of auxiliary transportation vehicles [1]. However, the underground environment is complex, and driverless auxiliary transportation vehicles need to be equipped with a reliable object-detection model to provide information for decision making [2,3].

**Figure 1:**
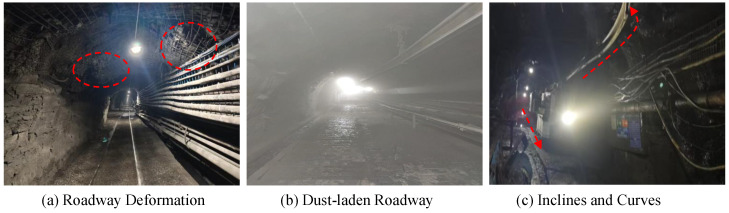
Complex perception scenarios.

The environment of underground mine tunnels is complex, as shown in Figure 1. The surrounding rock of the tunnels is prone to deformation due to ground pressure, posing safety hazards such as rock falls and roof collapses (Figure 1a), some areas have high dust and fog concentrations and uneven illumination (Figure 1b), and the tunnels have many slopes and curves (Figure 1c). Actual measurement data show that in auxiliary transportation vehicle operation areas, such as tunneling tunnels and return airways, dust mass concentration can reach up to 500 mg/m^3^, and most areas have a low illumination of approximately 5 lux. At the same time, under the influence of mining, the contraction ratio of some tunnel cross-sections can reach 23% [4,5,6].The above complex conditions result in poor data quality collected by sensors. The field of view of a single sensor is insufficient to fully cover the vehicle’s driving area, increasing the difficulty of sensing the underground environment and placing higher demands on the robustness and reliability of sensing methods.

Currently, multimodal fusion object-detection models based on BEV have achieved significant results in the field of autonomous driving. However, existing models are primarily applied to ground-based autonomous driving scenarios, where perception datasets are abundant and of high quality. When faced with multimodal data of uneven quality under the adverse conditions of underground environments, existing feature fusion strategies lack the ability to dynamically perceive the quality of modal data, making it difficult to ensure model robustness. Additionally, there is currently a lack of multimodal datasets for underground coal mines, limiting research and validation of multimodal fusion object-detection models for underground environments. Therefore, developing object-detection models tailored to underground coal mine environments faces dual challenges: feature fusion strategies that are not well-suited to the environment and a shortage of data resources.

To address these challenges, this paper proposes the Mine-DW-Fusion model based on multimodal fusion, which accurately recognizes and locates obstacles in underground coal mines by uniformly mapping image and point cloud data into BEV (Bird’s-Eye View) space. The main contributions of this paper are as follows:

- A Mixture of Experts-Fuzzy Logic Inference Module (MoE-FLIM) is designed to evaluate the confidence levels of BEV features from images and point clouds, enabling the dynamic allocation of multimodal feature weights.
- The Pyramid Multiscale Feature Enhancement and Fusion Module (PMS-FFEM) is proposed, which uses Gaussian pyramid multiscale decomposition of BEV features to enhance detail and coordinate features at different scales, achieving deep multiscale feature fusion.
- A multimodal perception dataset for underground coal mine environments is constructed, comprising camera images and LiDAR point clouds annotated with five typical obstacle types and four complex environmental scenarios, providing a foundation for model training and validation.

## 2. Related Work

### 2.1. Research on Target Detection in Coal Mine Shafts

Underground target-detection methods primarily focus on camera visual data and LiDAR 3D point cloud data. In terms of image target detection, research focuses on addressing image degradation issues caused by low illumination and high dust concentration in underground environments [7,8,9,10,11,12,13,14]. Ref. [15] addresses low-light conditions by proposing DK-YOLOv5, which improves the reliability of target detection in low-light environments by enhancing the SPPF layer and C3 module and incorporating the C2f-SKA attention mechanism; Ref. [16] improved YOLOv5s through data augmentation and attention mechanisms, proposing ODEL YOLOv5s suitable for harsh coal mine environments, thereby enhancing obstacle recognition accuracy for underground locomotives. In point cloud perception, the research challenge lies in the high point cloud noise caused by dust interference and rough rock surface conditions underground [17,18,19,20,21]. Ref. [22] uses a grid traversal method to filter the raw point cloud, then filters dust and fog noise points based on point cloud echo features before performing clustering detection; Ref. [23] proposes an improved Euclidean clustering algorithm that adaptively adjusts Euclidean clustering parameters based on distance and point cloud density, enhancing the robustness of underground point cloud target detection.

Although the aforementioned studies have improved the performance of single-modal object detection under specific conditions, single-modal data alone cannot fully describe the characteristics of underground environments. Image data lack depth information, and point cloud data cannot provide semantic information. Therefore, fusing multimodal data to achieve intermodal information association and complementarity is an effective approach to further enhance underground target-detection capabilities. Thus, this paper proposes Mine-DW-Fusion, which improves the accuracy of underground target detection by fusing multimodal data.

### 2.2. Target-Detection and Multimodal Fusion Methods Based on BEV Features

BEV feature encoding methods have emerged as one of the mainstream paradigms in object-detection technology for autonomous driving due to their unified spatial representation capabilities. In the field of visual detection, the Lift-Splat-Shoot method proposed in [24], maps monocular image data to the BEV space, implicitly extracting depth information from the image; Subsequently, methods such as BEVFormer [25], PolarFormer [26], and PETR [27] further enhanced BEV representation capabilities and model detection performance by introducing Transformer structures. In point cloud detection, Ref. [28] proposed the PointPillars method, which became a representative model for point cloud BEV object detection due to its real-time and efficient advantages; Ref. [29] further proposed CenterPoint, which integrated BEV encoding with an anchor-free detection mechanism, also demonstrating excellent detection performance.

With the rapid maturation of BEV feature-detection technology, research has gradually expanded from single-modal to multimodal fusion. The BEVFusion proposed in [30] is currently the most widely used multimodal fusion scheme, which uniformly encodes visual and point cloud features into the BEV space and then fuses them together. This approach has achieved significant improvements in object-detection performance compared to single-modal detection. Building upon BEVFusion, Ref. [31] introduces SimpleBEV, which incorporates cascaded depth estimation and LiDAR correction mechanisms. Additionally, by designing a camera-BEV feature-assisted detection branch and optimizing the multiscale sparse convolution feature fusion mechanism, the model’s ability to express and fuse multimodal features is further enhanced.

Although BEV-based object-detection models have achieved quite good results in recent years, existing research has mostly focused on ground scenes and has not addressed the special processing of low-quality underground data. Furthermore, the feature stitching fusion strategy is difficult to effectively cope with fluctuations in the quality of underground sensor data. In addition, due to the lack of underground multimodal object-detection datasets, existing results are difficult to directly apply to underground environments. To address these issues, this paper proposes the MoE-FLIM module, which dynamically allocates weights for different modality data, and constructs the PMS-FFEM module to deeply mine multimodal fusion features. Additionally, an underground multimodal dataset is constructed for model training and testing.

## 3. Methodology

**Figure 2:**
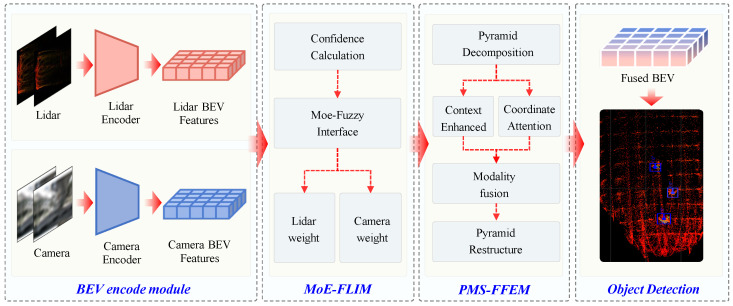
Mine-DW-Fusion overview. Multimodal inputs are processed separately by camera encoders and laser radar encoders, and fusion features are generated by MoE-FLIM and PMS-FFEM for target detection.

This section introduces Mine-DW-Fusion, a multimodal fusion object-detection model proposed based on BevFusion [30] for underground coal mine environments, which enhances detection performance through feature weighting and enhanced BEV fusion features. As shown in Figure 2, the model consists of a BEV feature encoding module (BEV Encoder), an expert mixture-fuzzy logic inference module (MoE-Fuzzy Logic Inference Module, MoE-FLIM), and a pyramid multiscale feature enhancement and fusion module (Pyramid Multiscale Feature Enhancement and Fusion Module, PMS-FFEM).

First, the raw data from LiDAR and camera are encoded into BEV features in a unified space. Then, the MoE-FLIM module is designed to assign fusion weights to different modal features through an expert-fuzzy logic inference mechanism. Next, the PMS-FFEM module is introduced, which uses a Gaussian pyramid for multiscale decomposition of features, and sequentially applies context enhancement and coordinate attention mechanism enhancement to generate BEV fusion features. Finally, the features are fed into five detection heads to perform detection of target category and position, target size, target angle, target height, and target center point offset, respectively.

The loss function in this study is defined as follows:

(1) $$L_{\mathrm{total}}=L_{\mathrm{center}}+L_{\mathrm{dims}}+L_{\mathrm{yaw}}+L_{z}+L_{\mathrm{xy}-\mathrm{delta}}$$

The loss function for object category and localization ($L_{\mathrm{center}}$) is computed using the improved Focal Loss, which effectively addresses the class imbalance between positive and negative samples in underground detection scenarios [32]. For object size ($L_{\mathrm{dims}}$), yaw angle ($L_{\mathrm{yaw}}$), height ($L_{z}$), and xy-delta ($L_{\mathrm{xy}-\mathrm{delta}}$), the loss functions are calculated using the Masked MSE Loss.

### 3.1. Lidar/Camera to BEV

**Figure 3:**
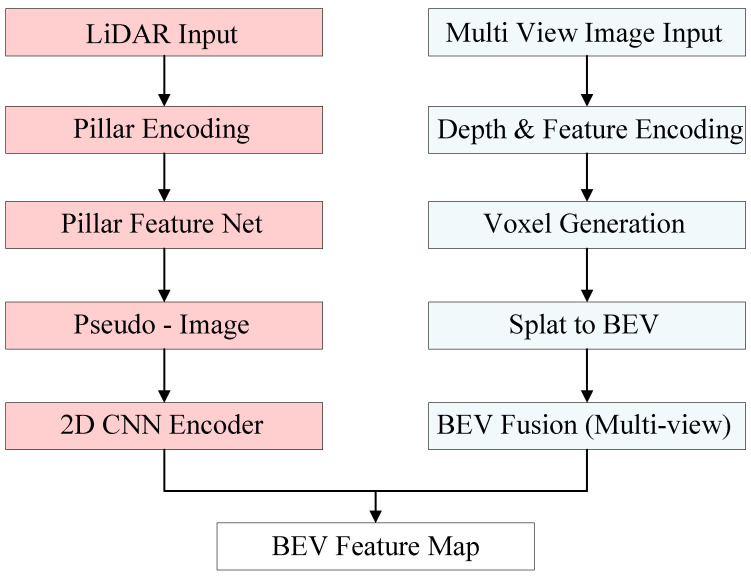
Technical route of BEV encoder.

To address the challenge of aligning multimodal features due to differences in perception fields and data dimensions, this paper transforms all modality data into a unified BEV feature map. BEV characterization is based on the vehicle body local coordinate system, which changes with vehicle motion. In this study, BEV feature encoding refers to bevfusion. Figure 3 illustrates the BEV feature encoding methods for LiDAR and camera modalities.

#### 3.1.1. Lidar BEV Stream

The PointPillars network [28] is used to perform BEV encoding of LiDAR point clouds. The main steps are as follows:

Step 1: Encode the point cloud as pillars. The features of each pillar are calculated based on the point cloud inside the pillar.

Step 2: Convert the pillar features into the top-view perspective, which completes the generation of the pseudo-image.

Step 3: Extract the 2D BEV feature map by applying 2D convolution.

#### 3.1.2. Camera BEV Stream

BEV features of the image are extracted using the LSS algorithm [24], with the following steps:

Step 1: Obtain the 3D features of each 2D pixel in space (LIFT). First, a D-dimensional depth space is predefined for the image to generate a D×H×W point cloud of view cones. Then, a context vector ($c\in R^{c}$) is predicted for each pixel *p* with coordinates (H, W), and the distribution α of each pixel over depth. This provides the context features at (D, H, W) as $c_{d}=\alpha_{d}c$.

Step 2: Splat. Based on the camera’s intrinsic and extrinsic parameter matrices, compute the 3D coordinates of the pixel in the principal coordinate system. The resulting 3D points are then assigned to the nearest Pillars. Summation pooling is performed to obtain a C×H×W tensor, and convolution operations are applied to this tensor to extract the image’s BEV features.

### 3.2. Mixture of Experts-Fuzzy Logic Inference Module

The quality of different modal data in coal mine shafts fluctuates with changes in the scene environment, leading to uneven data quality. In such cases, the feature concatenation method used by BevFusion is not effective in highlighting the contribution of effective features, limiting the model’s performance.

To address the above issues, this paper plans to adjust the weights of different features by calculating the confidence levels of different modal data. However, there is no clear mapping relationship between confidence levels and weight allocation. To model this uncertain mapping, this paper introduces fuzzy logic reasoning methods, using membership functions to divide confidence levels into continuous fuzzy states, and defining corresponding weighting rules to infer weights. Additionally, due to inherent differences among sensors, weight allocation varies when different sensor categories dominate. Therefore, this paper further integrates a multi-expert network mechanism, constructing multiple expert networks tailored to different sensor dominance scenarios to dynamically fuse expert outputs and achieve adaptive weight allocation in various scenarios. Thus, this paper proposes the Multi-Expert-Fuzzy Logic Inference Module (MoE-FLIM) to enhance model detection performance when sensor data quality is imbalanced.

**Figure 4:**
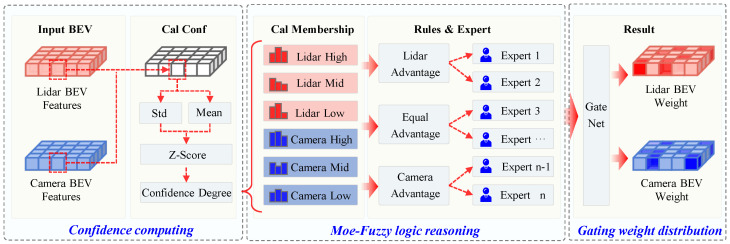
Diagram of the Mixture of Experts-Fuzzy Logic Inference Module.

As shown in Figure 4, we first calculate the Local Confidence Map for both LiDAR and camera BEV features, then map the confidence values to fuzzy states using the Membership Function and defining fuzzy rules. These fuzzy states are then evaluated by the corresponding expert network, and finally, a Gating Network is introduced to fuse the outputs from the expert networks and dynamically adjust the fusion weights of the sensor features.

#### 3.2.1. Confidence Calculation

Due to modality differences between the data collected by the camera and LiDAR, directly calculating the confidence on the raw data results in inconsistencies in units. Additionally, dimensionless processing of the results lacks theoretical support and is not sufficiently effective. To address this, the paper standardizes the confidence calculation for BEV features, as shown in (1). Given the sensor features ($x\in R^{B\times C\times H\times W}$), we first extract the C-dimensional feature vector at each spatial location. The local mean and standard deviation along the channel dimension are then computed to characterize the feature strength and stability. The results are subsequently normalized using Z-score and mapped through a Sigmoid function to obtain the confidence representation of the BEV features.

(2) $$Conf\left(x\right)=\mathrm{Sigmoid}\left(\frac{\mu (x)+\sigma (x)-E[\mu (x)+\sigma (x)]}{\sqrt{Var(\mu (x)+\sigma (x))}+\epsilon }\right)$$

where μ(x) is the mean mapping of the feature channel dimensions. The size is B×1×H×W, σ(x) is the mean and standard deviation of the local area characteristics and $\epsilon =1e^{-6}$, a small quantity constant to prevent division by zero.

#### 3.2.2. Moe-Fuzzy Logic Reasoning

**Figure 5:**
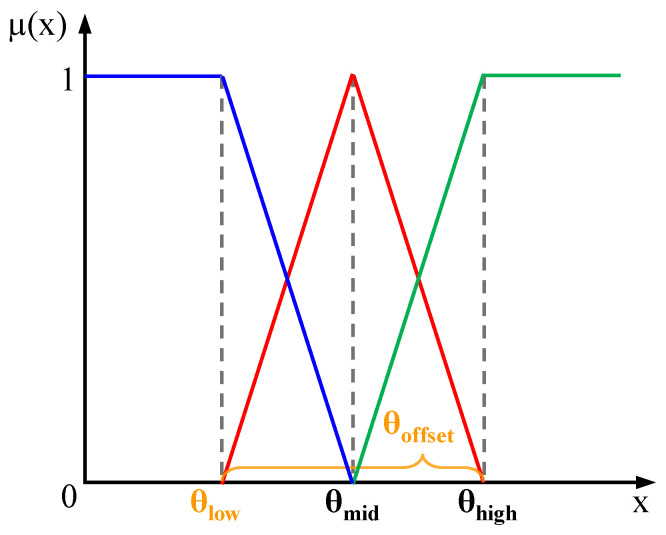
Triangular Membership Function.

Confidence is divided into three fuzzy states: low, medium, and high, which are defined by the triangular membership functions $\mu_{\mathrm{low}}\left(x\right),\mu_{\mathrm{mid}}\left(x\right),\mu_{\mathrm{high}}\left(x\right)$ to calculate the state values. The function images are shown in the Figure 5 below.

In order to ensure that the confidence threshold is more suitable for underground conditions, the confidence thresholds $\theta_{\mathrm{low}}$ and $\theta_{\mathrm{offset}}$ are set as learnable parameters. Through $\theta_{\mathrm{low}}$ and $\theta_{\mathrm{offset}}$, the expressions for $\theta_{\mathrm{mid}}$ and $\theta_{\mathrm{high}}$ are achieved, as shown in the formula:

(3) $$\theta_{\mathrm{high}}=\theta_{\mathrm{low}}+\theta_{\mathrm{offset}},\;\theta_{\mathrm{mid}}=\frac{(\theta_{\mathrm{high}}-\theta_{\mathrm{low})}}{2}$$

It should be noted that the reason for not directly defining the three thresholds as learnable parameters is to avoid the model losing the relationship $\theta_{\mathrm{low}}$ < $\theta_{\mathrm{mid}}$ < $\theta_{\mathrm{high}}$ during the learning process. The membership functions of the three fuzzy states are defined as:

(4) $$\mu_{\mathrm{low}}\left(x\right)=\frac{\theta_{\mathrm{mid}}-x}{\theta_{\mathrm{mid}}-\theta_{\mathrm{low}}},\mu_{\mathrm{high}}\left(x\right)=\frac{x-\theta_{\mathrm{mid}}}{\theta_{\mathrm{high}}-\theta_{\mathrm{mid}}},\mu_{\mathrm{mid}}\left(x\right)=1-\mu_{\mathrm{low}}\left(x\right)-\mu_{\mathrm{high}}\left(x\right)$$

The confidence level is mapped to a three-dimensional fuzzy membership vector using formula X. Based on the fuzzy state combinations of LiDAR and camera confidence, three sets of decision rules are defined: Equal, LiDAR Advantage, and Camera Advantage. By calculating and combining the membership product corresponding to the fuzzy state combinations, the fuzzy membership degree of LiDAR $\mu_{\mathrm{lidar}}\left(x\right)$ and the fuzzy membership degree of the camera $\mu_{\mathrm{camera}}\left(x\right)$ are obtained under each rule.

In traditional fuzzy logic systems, rule outputs are fixed. To enhance the expressive power of fuzzy reasoning, this paper configures multiple expert networks (Expert MLPs) for each rule, as shown in Formula (5). Each expert network takes the membership degree combination of LiDAR and camera data under that rule as input and outputs the weights of LiDAR and camera data under that rule.

(5) $$E\left(\mathrm{Rule}\right)=\mathrm{Softmax}\left(\mathrm{MLP}\left([\mu_{\mathrm{lidar}}\left(x\right),\mu_{\mathrm{camera}}\left(x\right)]\right)\right)$$

where MLP is a multilayer perceptron network. Each MLP consists of a fully connected layer and an ReLU activation layer.

#### 3.2.3. Gating Weight Distribution

A gating network is introduced to dynamically adjust expert network outputs. Its inputs comprise LiDAR confidence, camera confidence, their difference, and the absolute difference, enabling adaptive learning of spatial context. The gating network is defined as follows:

(6) $$ExpertGates=Softmax(\frac{GateNetwork(GateInput)}{T})$$

where GateNetwork is a network containing convolutional layers and nonlinear activation functions with an output dimension of the total number of expert networks, *T* is temperature parameter to regulate the sensitivity of the expert network weight assignment.

The weighted summation of the final expert network is the LiDAR feature fusion weights and the camera feature weights are also obtained.

(7) $$\begin{array}{cc} W_{\mathrm{lidar}},W_{\mathrm{camera}} & =\sum_{i=1}^{N}{ExpertGates}_{i}\times E_{i}\left(R\right) \end{array}$$

where num_experts is total number of expert networks, $W_{\mathrm{lidar}}$ is final LiDAR weights for feature fusion, $W_{\mathrm{camera}}$ is final camera weights for feature fusion.

### 3.3. Pyramid Multiscale Feature Enhancement and Fusion Module

**Figure 6:**
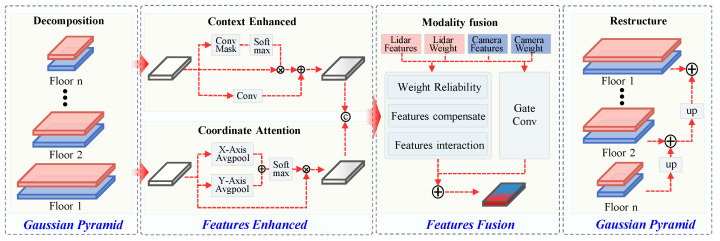
Diagram of the Mixture of Pyramid Multiscale Feature Enhancement and Fusion Module.

To address the issues of poor feature fusion quality and weak information expression caused by low-quality underground images and point cloud data, this paper proposes a multiscale feature enhancement and fusion module (PMS-FFEM) based on the Gaussian pyramid. This module primarily consists of multiscale decomposition and reconstruction using the Gaussian pyramid, as well as feature enhancement and fusion. The main workflow is illustrated in Figure 6. First, Gaussian pyramids are applied to decompose LiDAR and camera features into multiple scales. Then, at each scale, the features undergo contextual enhancement and coordinate attention enhancement, followed by fusion using reliability estimation and dynamic compensation strategies. Finally, a Gaussian pyramid is used to reconstruct the features across scales, resulting in a multiscale fused representation of the multimodal features.

#### 3.3.1. Multiscale Feature Decomposition and Reconstruction Based on Gaussian Pyramid

Previous studies have shown that the Gaussian pyramid structure has significant advantages in multiscale feature modeling and fine-grained target mining. Compared with conventional stride convolution or pooling strategies, it can more effectively retain low-level detail information and enhance high-level semantic features, thereby improving overall detection performance [33,34].

Given that different scales in BEV feature maps contain information of varying granularity, we use Gaussian convolution to perform layer-wise downsampling on the input feature maps, generating multilevel pyramid features. Given that different scales in BEV feature maps contain information of varying granularity, we use Gaussian convolution to perform layer-wise downsampling on the input feature maps, generating multilevel pyramid features. During the upsampling reconstruction process at each layer, information from different scales is gradually fused to enhance the overall contextual expression capability.

#### 3.3.2. Contextual Enhancement and Coordinate Attention Feature Enhancement

Contextual enhancement and coordinate attention enhancement are performed in parallel within each scale. The enhanced feature map and the weight information obtained from MoE-FLIM will be merged in subsequent steps.

Contextual enhancement: spatial adaptive pooling extracts global contextual information, which is then combined with the original features to enhance the features, as in (4):

(8) $$\begin{array}{c} F_{\mathrm{enhance}}=x\cdot O({\mathrm{Conv}}_{1\times 1}(\mathrm{Softmax}({\mathrm{Conv}}_{1\times 1}+{\mathrm{Conv}}_{1\times 1}\left(x\right) \end{array}$$

where *x* is the feature map with dimensions B×C×H×W, $Conv_{1\times 1}$ is 1×1 convolution operation, O(·) is context vectors after pooling and weighting spatial locations are expanded to their original size.

Define the fuzzy rule input based on the state combination of LiDAR and camera as follows:

(9) $$F_{\mathrm{att}}=\sigma \left(Conv_{1\times 1}\left(P_{H}\left(x\right)+P_{W}\left(x\right)\right)\right)$$

where $P_{H}\left(x\right)$ is features after pooling along the height direction (Y-axis), $P_{W}\left(x\right)$ is features after pooling along the width direction (x-axis), and σ is the Sigmoid activation function.

#### 3.3.3. Feature Fusion Based on Reliability Estimation and Cross-Modal Compensation

To deeply fuse features from different modalities, a feature fusion method based on modality reliability estimation and cross-modal feature compensation is proposed by exploiting the complementary perception capabilities between LiDAR and camera.

First, a convolutional network with dynamic weighting is employed to predict the local reliability of LiDAR and camera features, denoted as $r_{\mathrm{lidar}}$ and $r_{\mathrm{camera}}$, respectively. The predicted reliabilities are then corrected to obtain the final weights $W_{\mathrm{camera}}$ and $W_{\mathrm{lidar}}$ for the subsequent input.

Next, the compensated features are generated from the complementary modality features $F_{\mathrm{camera}}^{\prime }$ and $F_{\mathrm{lidar}}^{\prime }$, as follows:

(10) $$\begin{array}{cc} F_{\mathrm{lidar}}^{\prime } & =F_{\mathrm{lidar}}+C_{\mathrm{lidar}}\left(F_{\mathrm{camera}}\right)\cdot \left(1-W_{\mathrm{lidar}}^{\prime }\right) \end{array}$$

(11) $$\begin{array}{cc} F_{\mathrm{camera}}^{\prime } & =F_{\mathrm{camera}}+C_{\mathrm{camera}}\left(F_{\mathrm{lidar}}\right)\cdot \left(1-W_{\mathrm{camera}}^{\prime }\right) \end{array}$$

where C(·) generates a compensating gating factor via Sigmoid.

The compensated features are weighted by the corrected weights and fused to generate enhanced features:

(12) $$F_{\mathrm{fused}}={Conv}_{1\times 1}\left([F_{\mathrm{lidar}}^{\prime }\cdot W_{\mathrm{lidar}}^{\prime },F_{\mathrm{camera}}^{\prime }\cdot W_{\mathrm{camera}}^{\prime }]\right)$$

As shown in Formula (13), the original feature $F_{\mathrm{weight}}$ and enhanced feature $F_{\mathrm{fused}}$ are fused and weighted using a gating factor to obtain the final fused feature $F_{\mathrm{final}}$ at each scale. After feature reconstruction through the pyramid module, it can be used for object-detection tasks.

(13) $$F_{\mathrm{final}}=\mathrm{ReLU}\left(F_{\mathrm{fused}}+\mathrm{Gate}\left(F_{\mathrm{fused}},F_{\mathrm{weighted}}\right)\cdot F_{\mathrm{weighted}}\right)$$

where Gate(·) denotes the computational process of the gated convolution module that dynamically controls the ratio of the two fused features.

## 4. Experiments

### 4.1. Construction of the Multimodal Environmental Perception Dataset for Coal Mines

Current research in the field of multimodal environmental perception in underground coal mines is limited, and there is a lack of publicly available standard datasets. Existing datasets are insufficient to support the research of multimodal deep-learning models for coal mine underground environments. Therefore, this paper constructs a multimodal dataset specifically for auxiliary transportation scenarios in underground coal mines, covering typical roadway environments and complex perception conditions. This dataset provides fundamental data support for future research on multimodal environmental perception in underground coal mines.

**Figure 7:**
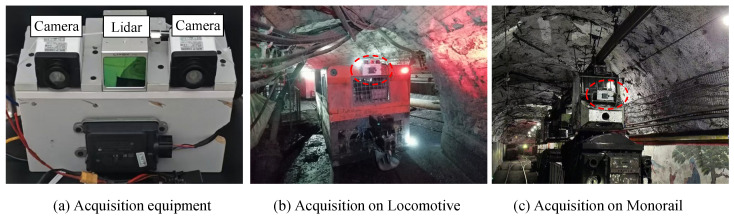
Multi-sensor data acquisition system and deployment.

The data acquisition equipment is shown in Figure 7 and consists of two RGB cameras, one laser radar, and one portable computing device. The image acquisition resolution is 640 × 480, and each frame of laser radar point cloud data contains approximately 24,000 points. The image and laser radar point cloud data are synchronized at the frame level through a unified timestamp. We employed three data acquisition methods: manual scanning, motor vehicle-mounted scanning, and monorail-mounted scanning. During data acquisition, the laser radar sampling frequency was set to 10 Hz, and the camera sampling frequency was set to 3 Hz. To prevent data loss, camera data timestamps were used to index and match laser radar data, ensuring alignment between radar and image data with an error margin of ±50 ms. Additionally, to synchronize the camera and LiDAR data for subsequent annotation, this study used the official Livox tool, Livox Camera-LiDAR Calibration Tool, for external parameter calibration. This involved capturing standard checkerboard calibration plates and collecting corresponding point cloud and image data. By combining image corner points with point cloud edge feature extraction, the PnP algorithm was used for initial estimation, followed by nonlinear least squares optimization to further refine the pose, ultimately achieving high-precision camera -radar external parameter calibration.

Coal mines feature numerous roadways with extensive coverage. This paper selects six representative areas for data collection, including the industrial site, shaft station, main haulage roadway, upper marshalling yard, inclined track haulage roadway, and return airway. These areas encompass various complex underground environments, such as low illumination, uneven lighting, and high dust and fog conditions.

**Figure 8:**
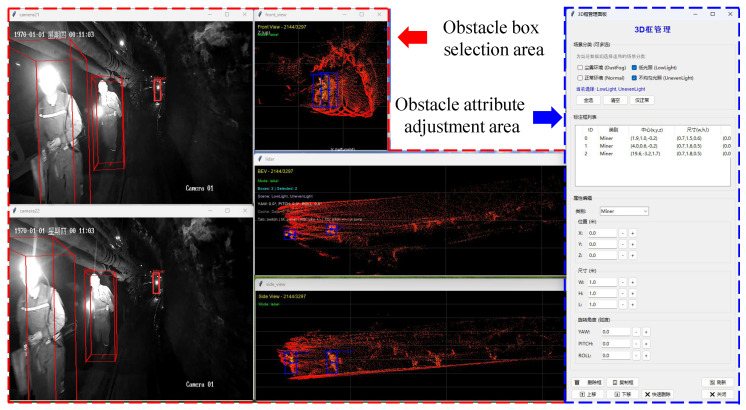
Data annotation software.

The dataset consists of a total of 3298 multimodal perception data sets, including 2632 sets for training and 666 sets for testing. It defines 5 types of typical obstacle targets: Miner, Mine Car, Device Box, Material, and Notice Board, as well as 4 types of typical environmental scenarios: Normal, Dust Fog, Low Light, and Uneven Lighting. As shown in Figure 8, we utilized Python to develop an annotation tool for the dataset, which captures location, category, size, and orientation information.

**Figure 9:**
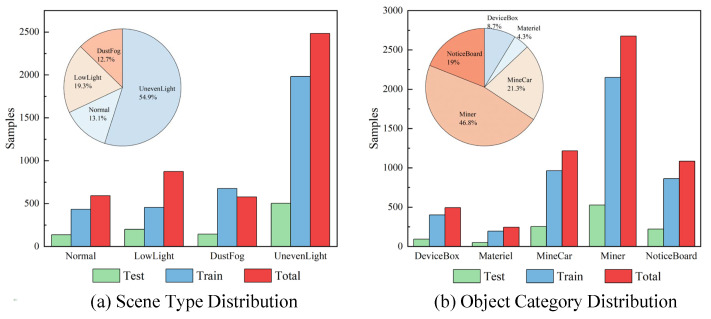
Dataset distribution.

The dataset distribution is shown in Figure 9. In terms of scene types, the underground environment is predominantly characterized by uneven lighting and low-light conditions, accounting for 54.9% and 19.3%, respectively. Normal scenes and dust and fog conditions together make up a smaller portion, accounting for 13.1% and 12.7%, respectively. Regarding target categories, the highest proportion is for Miner, accounting for 46.8%, reflecting the high occurrence frequency of miners in underground coal mine production scenarios. Miners are key objects in environmental sensing and safety monitoring. Next, Mine Car and Notice Board account for 21.3% and 19.0%, respectively. Overall, the dataset provides rich information on transportation and management scenarios, offering significant research and practical value.

### 4.2. Implementation Details

#### 4.2.1. Experimental Configuration

The image resolution for training and testing is 640 × 480, the maximum number of points per frame of the LiDAR point cloud is 24,000, and data enhancement methods such as cropping, flipping, and blurring adjustments are randomly performed during training. The batch size is set to 8, and the optimizer uses AdamW with a cosine annealing learning rate scheduling strategy with warm-up, a minimum learning rate of $10^{-4}$, and a warm-up duration of the first three epochs.The model is trained on a single A100 GPU. A total of 100 epochs are trained.

#### 4.2.2. Evaluation Indicators

The evaluation metrics mAP and NDS, which are commonly used for target detection, are introduced to comprehensively evaluate the model performance. Among them, mAP denotes the average value of precision rate under different recall rates. Since the self-built dataset does not contain speed and attribute information, the NDS metric is simplified as follows in this paper:

(14) $$\begin{array}{cc} NDS= & \frac{1}{10}(5\times mAP+\left(1-\min\left(1,\frac{ATE}{ATE_{\mathrm{norm}}}\right)\right)\\ & +\left(1-\min\left(1,\frac{ASE}{ASE_{\mathrm{norm}}}\right)\right)\\ & +\left(1-\min\left(1,\frac{AOE}{AOE_{\mathrm{norm}}}\right)\right)+2) \end{array}$$

where ATE (average translation error), ASE (average scale error), and AOE (average orientation error) reflect the accuracy of the predicted target in terms of position, size and orientation, respectively. The normalization constants: $ATE_{norm}=2.0$ m, $ASE_{norm}=0.1,$$AOE_{norm}=1.0$ rad. The two values of missing velocity error and attribute error are taken as 1.

### 4.3. Evaluation of Model Performance

In this section, the proposed method (Mine-DW-Fusion) is compared in detail with the current mainstream 3D detection algorithms. The methods are evaluated by retraining each model on the dataset, and Table 1 presents the evaluation results of different models on the test set.

The experimental results show that Mine-DW-Fusion outperforms other methods in both overall performance and category-specific detection accuracy. Compared to unimodal detection methods, Mine-DW-Fusion and BevFusion are able to fuse richer multimodal feature information, resulting in a substantial improvement in detection performance for both methods. In particular, Mine-DW-Fusion outperforms the suboptimal method BevFusion, with a 5.1% increase in mAP and a 2.6% increase in NDS. This indicates that the multimodal deep interaction fusion strategy proposed by Mine-DW-Fusion can more effectively fuse multimodal information, significantly enhancing the model’s overall detection capability.

Further analysis of the results in Table 1 reveals that, thanks to the MoE-FLIM module and the PMS-FFEM module, Mine-DW-Fusion demonstrates particularly significant advantages in the Miner and Notice Board categories. The MoE-FLIM module employs fuzzy logic reasoning and expert networks to dynamically allocate weights based on modal quality differences across various environmental scenarios, preserving the discriminative capabilities of superior modalities and significantly reducing the false negative rate for the Miner category. Meanwhile, the PMS-FFEM module enhances feature expression for small and medium-sized targets through multiscale feature decomposition, context enhancement, and cross-modal compensation, enabling targets such as Notice Board to maintain high detection accuracy even in complex backgrounds and low-contrast conditions. This mechanism of dynamic weight allocation and multiscale feature enhancement significantly improves the model’s overall detection capability.

**Figure 10:**
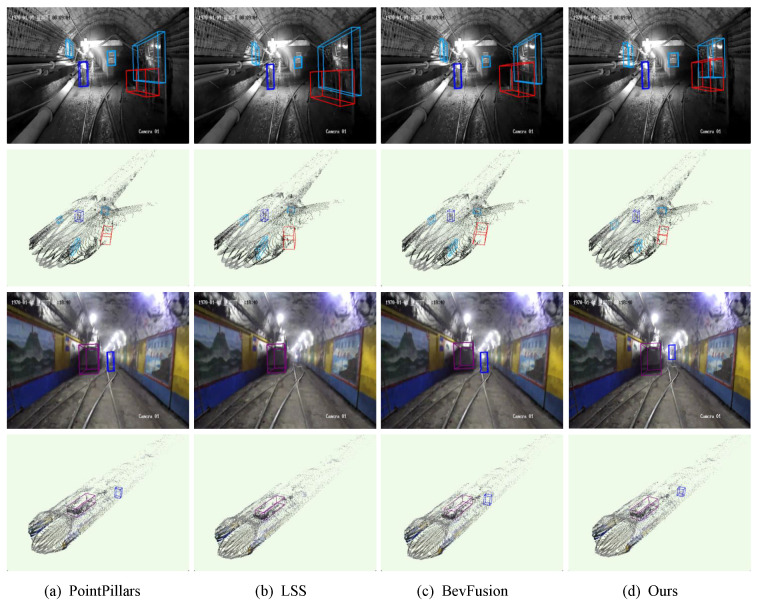
Detection effect of different target-detection methods in underground.

Figure 10 presents the performance of PointPillars, LSS, BevFusion, and Mine-DW-Fusion on the test set from both image and point cloud perspectives. The visualization results clearly demonstrate that Mine-DW-Fusion outperforms the other methods in mine scenarios. As shown in Figure 10b, the LSS method exhibits notable missed detections of personnel in the tunnel, whereas Mine-DW-Fusion accurately detects and locates various types of targets. Additionally, Mine-DW-Fusion demonstrates higher precision in detecting target sizes.

### 4.4. Model Performance in Typical Complex Environments of Coal Mines

To further assess the model’s perception capability in specific complex environments within coal mines, this paper classifies underground environments into three categories: dusty, low illumination, and uneven lighting. Specialized experimental evaluations of the model’s perception performance are conducted under each of these conditions.

The experimental results are shown in Table 2, where the proposed Mine-DW-Fusion method achieves the best detection performance under all three environmental conditions, demonstrating excellent adaptability and robustness. In low-light scenarios, Mine-DW-Fusion performs better than other models because LiDAR is not affected by lighting conditions. MoE-FLIM can dynamically increase the weight of the LiDAR modality to compensate for insufficient image information. Notably, in the dusty environment, the perception performance of all models shows a significant decline compared to the overall scene. This is primarily due to the severe attenuation of the LiDAR laser beam as it passes through the dust, leading to increased noise in the point cloud. Additionally, the dust substantially impacts the visibility of the camera images, significantly degrading image quality and, in turn, affecting the overall perceptual performance of the multimodal information. However, Mine-DW-Fusion still outperforms other models in dusty environments, indicating that PMS-FFEM can still extract and compensate for certain effective information even when sensors are simultaneously interfered with.

**Figure 11:**
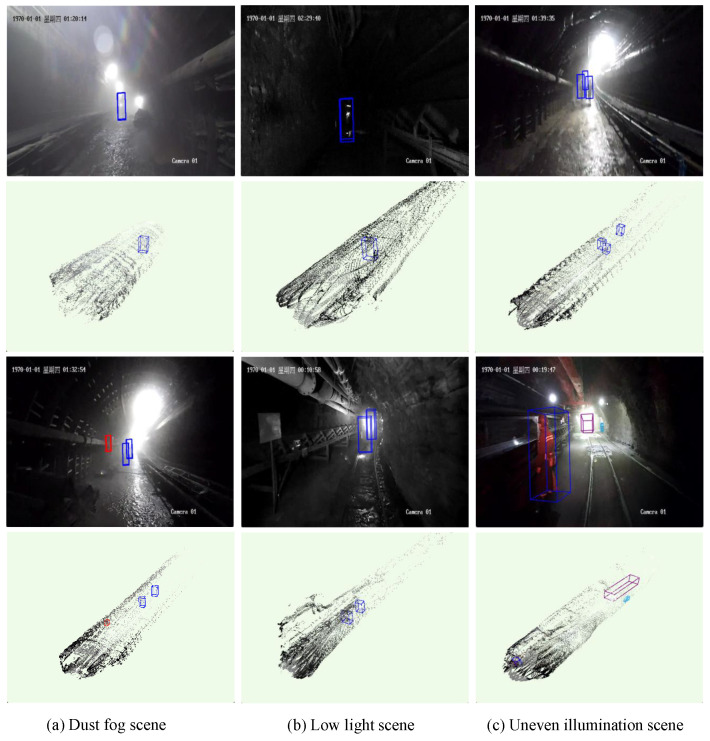
Detection effect of Mine-DW-Fusion method in different complex environments of coal mine.

Figure 11 shows the detection performance of the Mine-DW-Fusion method under the complex working conditions of a typical coal mine. The results indicate that the model can effectively recognize and locate personnel and equipment targets in low-light and uneven lighting conditions, exhibiting strong resistance to interference. Even in scenes with significant dust and fog, although the model’s detection performance decreases, it still maintains a certain level of personnel detection capability. These results suggest that the fusion of multimodal information by the Mine-DW-Fusion model yields promising performance even under challenging conditions.

### 4.5. Ablation Studies

This paper conducts extensive ablation experiments to assess the effectiveness of Mine-DW-Fusion. All experiments were performed using a custom test set.

#### 4.5.1. Mixture of Experts-Fuzzy Logic Inference Module

This section explores the optimal configuration of the MoE-FLIM. It analyzes the impact of the hidden layer dimension of the expert network, the number of experts corresponding to each rule group, and the gating temperature coefficient of the expert weights on the module’s performance.

Table 3 lists the impact of different expert network hidden layer dimensions on model performance. The experimental results show that as the hidden layer dimension increases from 4 to 16, the model performance improves consistently, with mAP and NDS increasing by 2.4 and 1.2, respectively. However, when the dimension is expanded to 32, overfitting occurs, with mAP and NDS decreasing by 4.8 and 2.4 percentage points, respectively. This indicates that a moderate increase in the hidden layer dimension can enhance model performance, but excessive expansion leads to significant performance degradation. When the hidden layer dimension exceeds 16, capacity oversaturation occurs.

Table 4 lists the impact of the number of experts assigned to each rule group on model performance. When each rule group is equipped with one expert, the model achieves baseline performance, with an mAP of 62.5 and an NDS of 68.6. When the number of experts per rule group is increased to two, the model’s performance improves significantly, with the mAP rising to 63.4 and the NDS to 69.0, indicating that the dual-expert strategy effectively enhances feature discrimination. However, when the number of experts is further increased to [3,3,3], performance declines markedly, with mAP and NDS dropping by 4.5 and 2.3, respectively. The performance remains below optimal levels even when the number of experts reaches [4,4,4]. The experiment confirms that more experts do not always lead to better performance. When each rule group is equipped with two experts, the model strikes the best balance between feature representation capability and parameter efficiency. Excessive numbers of experts result in model overcapacity and introduce decision noise.

Table 5 lists the impacts of different temperature coefficient settings on model performance. When the temperature coefficient reaches 1.0, the model achieves optimal global performance (mAP 65.4, NDS 70.3). At lower temperature coefficients (0.1 and 0.5), the performance of the two-expert system is poor, as overly sharp expert selection weakens the discriminative advantage of the multi-expert mechanism. The results indicate that model performance improves monotonically with an increase in the temperature coefficient, reaching its best performance at a coefficient of 1.0.

In summary, based on the results of the ablation experiments in this section, the optimal configuration for this module was determined to be a hidden layer dimension of 16, a rule expert assignment of (2, 2, 2), and a gating temperature coefficient of 1.

#### 4.5.2. Pyramid Multiscale Feature Enhancement and Fusion Module

The experiments in this section investigate the impact of the number of pyramid layers on model performance in the PMS-FFEM. The experimental results, shown in Table 6, reveal that when the number of pyramid layers increases from 1 to 2, the model performance remains relatively unchanged. However, when the number of layers increases to 4, the model performance improves further, with an mAP of 65.4% and an NDS of 70.3%, reaching its optimal performance. This suggests that a deeper hierarchical structure enhances the ability to model contextual information and express multiscale features. In contrast, when the number of layers is set to 3, model performance degrades significantly, likely due to feature imbalance caused by this configuration, which leads to a decline in the model’s expressive capability.

The ablation experiment results in this section show that a reasonable increase in the number of pyramid layers enhances model performance. Based on these findings, a pyramid layer count of 4 is determined to be the optimal configuration for this module.

#### 4.5.3. Module Validity

In the previous two sections, we conducted an in-depth parameter configuration analysis of the core modules in Mine-DW-Fusion to determine their optimal architectural settings. To further assess the actual contribution of each module within the overall architecture, this section performs a complete ablation of each submodule and conducts ablation tests under a unified configuration to evaluate its impact on final performance.

The experimental results are shown in Table 7. When only the MoE-FLIM is retained and the PMS-FFEM is removed, the model performance decreases significantly, with an mAP of 57.8% and an NDS of 66.2%. This indicates that the model’s performance is limited without the multiscale, in-depth fusion of multimodal features. When only the PMS-FFEM is retained and the MoE-FLIM is removed, the model’s performance remains lower than that of the full structure, with an mAP of 60.4% and an NDS of 67.6%. This shows that the MoE-FLIM plays a crucial role in improving feature discriminability. With the complete structure, the model achieves the best performance, with an mAP of 65.6% and an NDS of 70.3%.

The experimental results confirm that the synergistic combination of the two types of modules significantly enhances the model’s representational power and robustness. The modules proposed in this paper effectively improve the model’s detection performance.

### 4.6. Runtime Analysis

To further evaluate the computational efficiency of Mine-DW-Fusion, this paper sets the batch size to 1 and tests the model inference speed using FP32 precision on an NVIDIA A100 GPU (40 GB). The results are shown in Table 8.

Under the same hardware conditions, the inference speeds of PointPillars, LSS, and BEVFusion are 62.55 FPS, 42.61 FPS, and 27.06 FPS, respectively. while Mine-DW-Fusion achieves 9.54 FPS, with inference speeds significantly lower than other models. Our analysis found that PMS-FFEM consumes a significant amount of inference time, primarily due to the current optimal pyramid layer count of 4, which results in a large computational workload for multiscale feature analysis. While this improves detection accuracy, it also increases computational overhead.

In terms of GPU memory usage, the peak values for the four methods are 0.05, 0.09, 0.11, and 0.14 GB, respectively. As GPU memory usage increases, the inference speed of the model also slows down. However, the GPU memory usage of all four models is less than 0.1%, far below the device limit, indicating that GPU memory is not a performance bottleneck, and the differences in inference speed primarily stem from computational complexity. Overall, Mine-DW-Fusion improves the accuracy of underground target detection at the cost of some real-time performance. The operating speed of underground auxiliary transport vehicles is generally around 1.5–2.5 m/s, and the current model can only achieve near real-time perception in low-speed driving scenarios. In the future, we will optimize the PMS-FFEM module and further enhance the model’s detection speed through pruning distillation and TensorRT acceleration to meet the real-time inference requirements of engineering applications

## 5. Conclusions

In this paper, Mine-DW-Fusion is proposed to address the multimodal perception problem in the complex environment of underground coal mines. The model introduces the MoE-fuzzy logic inference method for dynamic allocation of multimodal feature weights and combines it with a pyramid multiscale enhancement fusion strategy to perform feature enhancement and fusion at different scales. Additionally, this study constructs a multimodal environment perception dataset for underground coal mines, and Mine-DW-Fusion is trained and tested on this dataset. The experimental results show that, in terms of overall performance, Mine-DW-Fusion achieves an mAP of 65.6% and an NDS of 70.3%. Under typical harsh conditions such as dust and fog, low illumination, and uneven lighting, the model achieves mAP values of 51.1%, 79.0%, and 65.3%, respectively, demonstrating strong environmental adaptability. The ablation experiments confirm that the proposed MoE-FLIM and PMS-FFEM contribute 7.8% and 4.8% improvements in mAP, respectively.

Mine-DW-Fusion is one of the few existing multimodal environmental perception methods for underground environments. We hope this research will provide a more effective and feasible technical approach for environmental perception in underground autonomous driving scenarios. While achieving the aforementioned results, we also recognize that this study has room for further improvement in several areas. First, the current model is primarily designed to address issues of sensor quality imbalance and has not yet fully considered extreme conditions where sensor damage leads to complete data loss. Second, while the constructed dataset covers typical driving scenarios for underground auxiliary transport vehicles, the data were primarily collected from mines in southwestern China. The model’s robustness and generalization capabilities in cross-domain adaptability across multiple mining areas, especially in out-of-distribution environments, require further validation. Additionally, this work focuses on model design, and further research is needed on the deployment and engineering implementation of the model in real underground environments.

Future work will focus on reducing computational overhead while maintaining detection accuracy through methods such as model lightweighting, inference acceleration, and knowledge distillation, while further expanding the coverage and diversity of the dataset. At the same time, we will introduce robustness optimization strategies for sensor failure and out-of-distribution inputs to improve the stability and reliability of the model in a wider range of practical application scenarios.

## Figures and Tables

**Table 1:** Comparative experiments with mainstream detection methods on our custom dataset.

Method	Modality	mAP	NDS	Miner		Device		Noticeboard		MineCar		Materials	
				AP	NDS	AP	NDS	AP	NDS	AP	NDS	AP	NDS
Pointpillars [28]	L	56	65.2	75.1	73.9	41.3	58.9	46.4	61.5	87.6	81.9	29.4	52.7
LSS [24]	C	47.9	61.2	57.7	65.8	41.1	58.7	39	57	75.8	75.6	25.8	49.9
BevFusion [30]	L + C	60.5	67.7	72.1	72.8	54.9	65.7	43	59.7	87.6	81.9	45	60.9
Mine-DW-Fusion	L + C	**65.6**	**70.3**	**79.9**	**76.7**	**60.5**	**68.6**	**52**	**64.3**	**89.4**	**82.8**	**46.3**	**61.2**

**Table 2:** Mine-DW-Fusion detection performance in different complex environments.

Method	DustFog		LowLight		UnevenLight	
	mAP	NDS	mAP	NDS	mAP	NDS
pointpillars	43.1	58.8	48.5	61.1	56.8	65.8
LSS	24.8	49.5	47.5	61	48.7	61.6
BevFusion	49.2	62.1	61.8	68.1	61.1	68.2
Mine-DW-Fusion	**51.1**	**62.9**	**79**	**76.7**	**65.3**	**70.2**

**Table 3:** Impact of hidden layer dimensions in expert networks.

Hidden Layer Dimension	mAP	NDS
4	60.1	67.4
8	61.7	68.3
16	**62.5**	**68.6**
32	57.7	66.2

**Table 4:** Effect of number of experts on model performance.

Numbers of Experts	mAP	NDS
1, 1, 1	62.5	68.6
2, 2, 2	**63.4**	**69**
3, 3, 3	58.9	66.7
4, 4, 4	59.1	67

**Table 5:** Effect of gating temperature coefficient on model performance.

Temperature	mAP	NDS
0.1	59	66.9
0.5	58.3	66.5
1	**63.4**	**70.3**

**Table 6:** Effect of pyramid layer count on model performance.

Pyramid Layers	mAP	NDS
1	63.8	69.5
2	63.4	69
3	58.3	66.5
4	**65.6**	**70.3**

**Table 7:** Ablation results of core modules.

Module		mAP	NDS
MoE-FLIM	PMS-FFEM		
✔		57.8	66.2
	✔	60.4	67.6
✔	✔	**65.6**	**70.3**

**Table 8:** Ablation results of core modules.

Method	FPS	Avg. Inf. Time (s)	Std. of Inf. Time (s)	Memory (GB)	Mem. Util.
Pointpillars	62.55	0.016	0.0237	0.05	0.125%
LSS	42.61	0.0235	0.0518	0.09	0.225%
BevFusion	27.06	0.0369	0.0365	0.11	0.275%
Mine-DW-Fusion	9.54	0.1048	0.0413	0.14	0.350%

## Data Availability

Some or all data, models, or code generated or used during the study are proprietary or confidential in nature and may only be provided with restrictions.
